# Crosstalk between triple negative breast cancer and microenvironment

**DOI:** 10.18632/oncotarget.28397

**Published:** 2023-03-31

**Authors:** Karly Smrekar, Artem Belyakov, Kideok Jin

**Affiliations:** ^1^Department of Pharmaceutical Sciences, Albany College of Pharmacy and Health Science, Albany, NY 12208, USA

**Keywords:** triple negative breast cancer, tumor microenvironment, current therapy

## Abstract

Although many advances have been made in the treatment of breast cancer, for the triple negative breast cancer (TNBC) these therapies have not significantly increased overall survival. Tumor microenvironment (TME) plays an essential role to develop and control TNBC progression. Many preclinical and clinical studies are ongoing to treat patients with TNBC disease, but the effective therapies are currently not available. Here, we have reviewed recent progress in understanding of TNBC and advance in defining mechanisms of TNBC therapies and potential therapeutic strategies to overcome TNBC.

## INTRODUCTION

Technological advancements such as genomics and epigenomics have provided us with vast insight about the complexity of breast cancer. However, one thing has remained the same, the need for the evaluation of three markers. These three markers; the expression of estrogen, progesterone, and HER2, are all molecular targets for treatment regimens, and are relied on by clinicians [1]. Chemotherapy is the staple treatment for TNBC patients. However, they lack the expression of three key therapeutic markers. The lack of therapeutic markers leads to poorer outcomes in TNBC.

TNBC is a tumor with heterogeneous behavior and has been labeled as “inherently aggressive” [2]. Histologically, TNBC can be classified as an invasive carcinoma with the majority having no subtype. General features include harbor pushing borders, brisk lymphocytic infiltrates, areas of necrosis, and medullary features such as syncytial growth and metaplastic elements [3].

Initially, TNBC was differentiated only using receptor status as criteria [4]. Recent work has been done to distinguish TNBC subtypes transcriptionally. Burstein et al. had separated TNBC into 5 distinct groups using RNA and DNA profiling analysis. These groups are composed of the following: LAR, MES, BLIS, BLIA [5]. LAR was characterized by downregulated cell cycle components, upregulated immune signaling with immune related death pathways, have intact AR, ER, prolactin and ErbB45 signaling, and expressed ESR1 and other estrogen related genes despite being ERalpha- on immunohistochemistry analysis [5]. MES was found to also have downregulated cell cycle components and upregulated immune signaling and immune mediated death pathways, but uniquely has upregulated osteocyte gene (OGN), adipocyte genes (ADIPOQ, PLIN1) and growth factors (such as IGF1) [5]. The BLIS subgroup was noted to have a lack of p53 gene activation, downregulation of B-, T-, and NK cell regulating pathways as well as downregulation of cytokine pathways and a unique expression of multiple SOX family transcription factors [5]. The BLIA subgroup has as well a lack of p53 gene activation, and interestingly highly expressed and activated STAT genes and upregulation of B-, T-, and NK cell function regulators [5].

Burstein’s BLIS and BLIA subtypes correspond to the previously found subtype of basal-like breast cancer cells. BLBC cells express gene characteristics of normal basal myoepithelial cells [6]. Even with the similarities, there is still much difference between these and other subtypes, and even within BLBC, there appears to be a rift on how these cancers interact with immune components of the tumor stroma [3, 5, 6]. Although acknowledging that TNBC is markedly heterogenous, there is still more work needed for experimental and clinical applications of these groupings.

Mutation rate in TNBCs are about 1.68 somatic mutations per Mb of coding region but can reach as high as 4.68 somatic mutations per Mb [7, 8]. The most frequent mutations are TP53 mutations and are more common in basal TNBC compared to non-basal TNBC. However, TNBC’s ability to be driven by aberrations is very low, and this presents a challenge for drug development.

The BRCA1 and BRCA2 genes encode proteins critical for maintaining DNA integrity and genomic stability [9, 10]. In the presence of a germline mutation in either BRCA1 or BRCA2, a person’s lifetime risk of breast cancer increases by 60–70%. Specifically, in BRCA1/2 proficient TNBC, the overexpression of genes such as ID4 or HORMAD1 can be a potential driver of genomic instability and BRCAness [1]. Those who get breast tumors from carrying BRCA1 mutations have basal-like features (high frequency of TP53 mutations) and a question that should be further explored is if the loss of BRCA1 can cause the pathogenesis of BLBC and TNBC because of defective DNA-repair pathways.

## TUMOR MICROENVIRONMENT (TME)

Cancer does not exist in a vacuum. The stroma interacting with tumors may include cells such as fibroblasts, epithelial cells, macrophages, T-lymphocytes, dendritic cells, neutrophils and adipocytes as well as structural components such as lymphatic and blood vessels and soluble factors such as growth factors, cytokines and chemokines [11, 12]. The stroma typically serves as an antitumor barrier but can transform into a tumor promoting state. This change can be intrinsic such as inefficient vasculature or can be acquired such as reactivity to chemo- and radiotherapy mediated through fibroblasts and immunosuppressive cells [11]. The roles of separate TME components in tumor promotion will be discussed in the following sections.

### Fibroblasts and tumor vasculature

Normally, fibroblasts maintain the structural framework in tissues, and suppress tumor formation [13, 14]. These fibroblasts can be characterized by expression of NFkB and TGFbeta in wound healing and inflammatory states and generally can be recognized using the marker Fibroblast Activation Protein [14].

Cancer associated fibroblasts are less well characterized than their nontumor version. Cell origins of fibroblasts are still being investigated but common markers for this cell subtype include HSF1, STAT3, MYC and YAP [14].

Further phenotypic and functional heterogeneity of both types of fibroblasts exist and is reflected in alterations in response to damaged tissue for remodeling [15, 16]. These differing responses can be implicated to the unique damage signals (including cytokines, chemokines and cell components) that fibroblasts become exposed to [17, 18]. Understanding of stromal fibroblast inter- and intratumoral heterogeneity is impaired by the lack of specific markers. However, it has been shown for CAFs within tumors to have a correlation between an abundance of stromal cells and poor prognosis, and vice versa [19].

Cancer associated fibroblasts are increasingly shown to have a complex relationship in promoting tumor survival, growth, and proliferation.

The development of a vascular network in a tumor can limit the tumor growth. Vascular networks are derived through angiogenesis (new blood vessels form from existing ones) and vasculogenesis (blood vessel formation via production of endothelial cells) [11]. Poorly organized tumor vasculatures due to uneven vasculature of differing maturity, and decreased drainage from poor lymphatic vessel coverage often have a hypoxic environment and have a limited nutrient supply [11]. As well, alterations in vasculature can create areas of a tumor that receive varying amounts of drugs that have to diffuse out of systemic circulation leading to impacts on tumor heterogeneity and clinical outcomes [20].

### Immune cells and the immune system

The immune system typically plays the role of tissue protection in cases of infection and tissue damage. The immune system exists as well as a barrier to tumorigenesis by detection of premalignant and malignant tissue but immunosuppression can dampen the response [11].

The complex interaction of cancer cells and immune system components has shifted from just immune surveillance but to immunoediting [21, 22]. Immunoediting serves to characterize both the host protection component as well as tumor sculping whereas the immune status of the tumor microenvironment presents three distinct phases of this interplay [1]. The elimination phase corresponds with the immune surveillance function, the equilibrium phase engages the immune system to keep the tumor under control, and the escape phase corresponds to cells which breached the host organisms’ immune defenses [23].

### Tumor associated macrophages (TAMs)

Macrophages are both antitumoral and pro-tumoral. In cancer-initiating conditions macrophages are anti-tumoral, whereas once the tumor is established the macrophage becomes tumor promoting [24]. Alterations of the macrophage phenotype occurs in all steps of the establishment of a tumor, with initiation, progression and metastasis. Macrophage subtypes of TAM, including M1 and M2 have been linked with supporting tumor growth via angiogenesis, tissue remodeling as well as general suppression of antitumor immune responses [25].

Macrophages act as a critical component of the tumor microenvironment with in some cases composing up to 50% of the tumor mass and have been found to be an independent association of poor prognosis in most cancers [26–29]. The TME of metastasis which contains macrophages among several other components, has been shown to be predictive of metastatic potential in breast cancer [30]. This is because the TAMs show delayed and defective NF-*Κ*B activation in response to signals and so the TAMs sustain flaring inflammation in the TME, resulting in protumor phenotypes [31]. The activation of TAMs can be reversed by IFN-γ and new strategies using the identification of genetic and epigenetic mechanisms of the macrophage are being used to target the reeducation of the TAM [31–33].

## CURRENT THERAPIES

Chemotherapy has been the mainstay treatment for TNBC for over 20 years. However with TNBC patients, chemotherapy is known as the “TNBC paradox” where patients have a high recurrence rate on no treatment but also have risk of receiving little therapeutic benefit from treatment [34].

Additionally, TNBC tends to respond best to dose-dense and high-dose regimens. Even with a higher response with chemotherapy, end results are often dismal. Survival 5 years post diagnosis is seen in less than 30% of patients and metastatic TNBC results in death for nearly all women afflicted [35].

**Figure 1:**
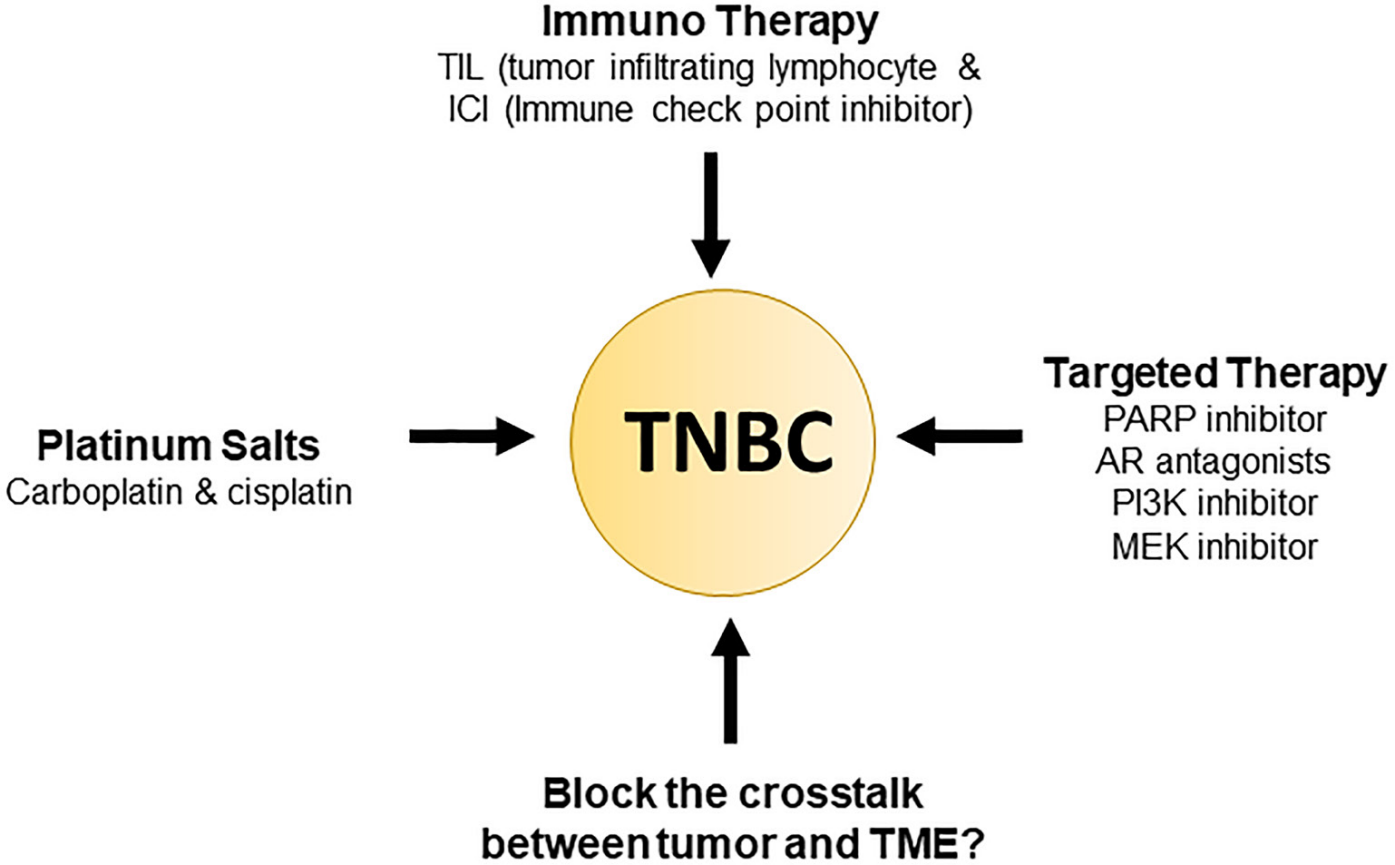
Current triple negative breast cancer therapy.

**Figure 2:**
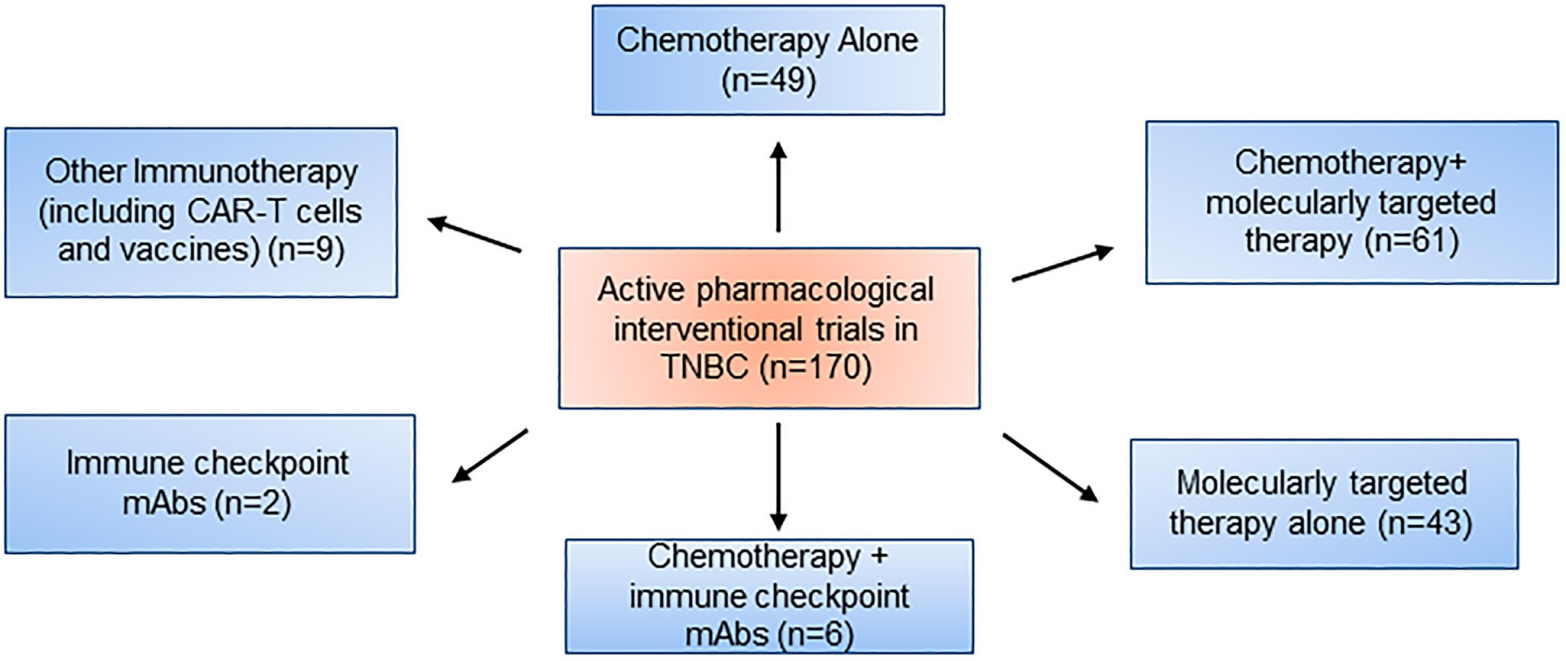
Active pharmacological intervention trials in TNBC.

Therefore, to manage patients properly, treatment approaches need to focus on a balance of current therapeutics and the newfound molecular complexity of TNBC as previously mentioned (Figure 1). The ESMO and ASCO guidelines further back up this point by a recommendation of usage of “sequential single-agent chemotherapy” excluding clinical cases involving visceral crisis or rapid progression of disease state. Additionally, as of 2015, 170 pharmacological interventional trials were being conducted, and a breakdown can be seen in Figure 2 [36, 37].

### Platinum salts

Currently clinical trials being done on platinum agents and their use in TNBC. Mechanistically, platinum salts, such as carboplatin and cisplatin, target cancer by activating apoptotic signaling pathways due to DNA crosslinks and resulting strand breakage. TNBC is especially sensitive to DNA damaging agents due to its inherent defective DNA repair machinery [38]. A single arm multicenter phase 2 trial showed that in 86 metastatic TNBC patients, there was a 32% overall response rate (ORR) to cisplatin and a 19% ORR to carboplatin [39]. Mutated BRCA1/2 patients had a higher ORR. This trial showed that there was a modest response to platinum-based agents. Several trials examined the neoadjuvant treated patients using platinum-based agents and assessed pCR rate as the endpoint. The findings revealed that approximately 30% of women who received anthracycline and taxane-based chemotherapy achieved pCR after therapy [35]. Among TNBC patients, those who achieved pCR after neoadjuvant chemotherapy had better disease-free survival rates compared to those who did not achieve pCR. In a 51-patient trial that used neoadjuvant cisplatin and anti-VEGF antibody bevacizumab, the pCR rate was 15% [40]. These results suggest that platinum-based agents can be effective either as single agents or as additions to neoadjuvant regimens in early stage TNBC patients. However, the addition of platinum-based agents remains controversial due to the possibility of toxicity and the lack of correlation between improved pCR and event survival. The GeparSixto trial showed a sub sequentially increased toxicity, and many patients were unable to complete the trials [41]. The CtNEO meta-analysis also failed to demonstrate a correlation between improved pCR and improved event survival.

### Targeted therapy

Cases of TNBC that persist after failed chemotherapy still have other targetable antitumor pathways, with as much as 90% of persistent cases having alterations that may be exploited with investigational agents [42]. The use of personalized treatment strategies which would target the molecular tumor-specific alterations would be able to effectively treat 60–70% of TNBC patients who do not respond to chemotherapy [1]. PARP, anti-androgen therapy, PI3K inhibitors, and MEK inhibitors are prime examples of targeted therapy, and their mechanisms will be explained in the upcoming sections.

### PARP

PARP is a nuclear enzyme that is abundantly and constitutively expressed. It is involved in targeting proteins, facilitating DNA repair, and signaling to critical cell-cycle proteins and oncogenes. This is achieved through the catalysis of ADP-ribose transfer from NAD+ [43]. Inhibition of PARP is a target in cancer treatment because its inhibition via RNA interference or chemical inhibitors can result in double-stranded breaks in replicating cells, leading to highly selective toxicity in BRCA1 and BRCA2 defective tumors [44, 45]. PARP inhibitors serve several roles in cancer treatment, including sensitization to chemotherapy and radiotherapy, synthetic lethality in tumors from patients with hereditary mutations in BRCA1/2 genes, and leveraging of supposed “BRCA-like” defects and defects in DNA repair [44, 46].

PARP inhibitors such as Olaparib, veliparib, and rucaparib are currently undergoing testing. Olaparib has shown significant single-agent activity in BRCA-deficient patients with response rates ranging from 22% to 41% with minimal toxicity at 100 mg twice a day and 400 mg twice a day, respectively [47]. Veliparib, when added to standard chemotherapy regimens with carboplatin for patients with stage II or III TNBC, resulted in increased pCR rates from 26% to 52% [48]. However, similar results were obtained with just the addition of carboplatin, making it unclear whether veliparib has a positive correlation. Finally, the use of cisplatin with or without rucaparib in patients showed that disease-free survival at 1 year was similar in both treatment groups, with rucaparib not adding any substantial effect [49]. These results demonstrate that while PARP inhibition alone has benefits, combination therapy with platinum agents has unclear benefits.

### Anti-androgen therapy

Interestingly, despite the altered estrogen signaling pathway giving the impression that TNBC is unaffected by hormone signaling, targeting androgen signaling is a promising avenue for therapeutic response using targeted hormone therapy, at least in AR-positive TNBC. Experimentally, the LAR subtype of TNBC has been susceptible to AR antagonism both *in vitro* and *in vivo*. Bicalutamide, an androgen-blocking agent was evaluated in a phase II trial and showed clinical benefit in 19% of patients at six months [50]. LAR-subtype cell lines are also enriched with PIK3CA activating mutations. They exhibit strong sensitivity to PI3K inhibitors and androgen blockers so the combination of PI3K inhibitors with an androgen blocker is being explored as a possible target in AR-positive TNBC [51, 52].

### PI3K inhibitors

The most common activating mutations in TNBC affecting the PIK3CA catalytic subunit α are PIK3CA mutations [8]. PI3K inhibitors are relevant in chemotherapy because they regulate cell growth, metabolism, and survival, and also stabilize double-strand breaks and create a BRCA1/2-like deficient state by interacting with the homologous recombination complex [53]. PI3K inhibition is particularly useful in combination with PARP inhibition because it downregulates BRCA1/2, creating a BRCA-mutant-like tumor state and sensitizing BRCA1/2-proficient tumors to PARP inhibition [54].

### MEK inhibitors

TNBC cells are responsive to MEK inhibition *in vitro*, as they are influenced by the Ras/MAPK pathway that is facilitated by MEK inhibitors [55]. Nonetheless, several TNBC cell lines demonstrate upregulation of the Ras/MAPK pathway, despite the absence of an oncogenic mutation. This can be attributed to the activation or overexpression of growth factor receptors, or gene copy-number alteration, which lead to increased gene expression [56, 57].

The c-Myc oncogene is amplified in approximately 30% of patients with TNBC or BLBC [56, 58], making it an important therapeutic target. MEK inhibition can lead to the degradation of c-Myc, but this also triggers the expression and activation of receptor tyrosine kinases, which can promote therapy resistance by bypassing MEK inhibition [59]. Therefore, combination of MEK inhibitors with small molecules or monoclonal antibodies which target receptor-tyrosine kinases are currently being validated, and combinations with chemotherapy and other targeted TNBC and BLBC agents are under investigation. Importantly, orally available potent inhibitors of MEK ½ (gemcitabine and trametinib) are being used in patients with solid tumors in a phase 1b trial, and the only complete response to therapy occurred in a mTNBC patient [59].

### The new age of immunotherapy and future clinical direction for TNBC

The immune system has always played a role in cancer, but only recently has immunotherapy become a major tool for cancer treatment with unprecedented activity and possibility for cure [60–64].

It was previously established that carcinogenesis is due to a deficiency of immune surveillance and that idea had been the basis for immune therapy. Current directions for therapies are the development of targeting immune checkpoints to augment the body’s pre-existing immune response for a more sensitive, stronger, and broader anti-cancer immunity [65, 66].

Preclinical studies found that there exists a great synergism between chemotherapy and immune system function to target tumor tissue in TNBC [67, 68]. As well, there is a correlation between immune marker expression and benefit found from chemotherapy for TNBC. However, exact clinical impacts from drug specific immunomodulation is unknown remains a topic needing more investigation.

A characteristic of TNBC includes a greater presence of tumor infiltrating lymphocytes (TILs) as well as greater expression of programmed cell death ligand 1 (PD-L1) when compared to other subtypes of breast cancers [69–73]. PD-L1 plays a role in regulating immune tolerance, and the primary mechanism of PD-L1 regulation in TNBC is the development of resistance to immune responses [1]. Aside from acquired resistance, PD-L1 expression can also be influenced by molecular alterations and oncogenic pathways [1]. For instance, PTEN deficiency in TNBCs is linked to the overexpression of PD-L1, thus supporting the association between elevated PI3K signaling and the presence of PD-L1 [72, 74].

A person with a “hot” immune system, or one that contains a higher TIL presence, is considered to have a better prognosis and higher likelihood of benefit from chemotherapy [67, 75]. Therefore, high levels of TILs equate to low risk of relapse and/ or death in early-stage TNBC patients treated with chemotherapy. These results suggest that by adopting the appropriate immune markers for risk stratification we can stratify patients by risk of recurrence, and patients which show low TIL presence have a higher risk of relapse and are a high priority [1, 76].

Immunotherapies are currently being tested in phase 1 trials in the TNBC population, because of the strong rationale presented above. A clinical trial was conducted to treat patients with advanced TNBC stage who showed positive expression for PD-L1 with pembrolizumab, a monoclonal antibody targeting PD-1. There was an 18.5% response rate [52]. In the other trial, the response rate was similar, with an ORR of 19% [77]. These two trials have brought about the problem of assay heterogeneity and showed how standardization and harmonization of PD-L1 testing is a major issue and a major goal.

This is a set back from the promises seen in preclinical models regarding immune checkpoint inhibitors and the extrapolated curative potential but more trials are ongoing to elucidate the potential of multifactorial chemotherapy and immune checkpoint inhibitor regimens in the neoadjuvant setting [78].

Overall, with better understanding of the interactions between cells in the tumor microenvironment come the discovery of druggable targets to take advantage of the molecular and immunological aspects of TNBC. The immunotherapy in TNBC is still in the early stage, which is challenging to take an advantage for a large population of TNBC patients and escalate the efficacy of immunotherapy. Furthermore, the side effects, toxicity, duration, dosage, and sequence of immune therapy should be considered to improve the clinical outcome. Currently, many preclinical and clinical studies are ongoing to discover the best combination of immunotherapy with other therapies. Consequently, we predict that the optimal combination strategy with high efficacy could be selected for TNBC patients. Moreover, we should consider for the resistance to immunotherapy and understand a mechanism to discover the potential biomarkers for predicting the efficacy of immunotherapy. Again, the study of immunotherapy for treating triple negative breast cancer might still be at its early stages of development but is full of future promise.
