# Addendum: Crosstalk between triple negative breast cancer and microenvironment

**DOI:** 10.18632/oncotarget.28507

**Published:** 2023-09-15

**Authors:** Karly Smrekar, Artem Belyakov, Kideok Jin

**Affiliations:** ^1^Department of Pharmaceutical Sciences, Albany College of Pharmacy and Health Science, Albany, NY 12208, USA

**This article has an addendum:** Research funding was obtained from the Albany College of Pharmacy and Health Sciences, Scholarship of Discovery Fund.

Original article: Oncotarget. 2023; 14:284–293. 284-293. https://doi.org/10.18632/oncotarget.28397

